# Rumen-protected methionine during the peripartal period in dairy cows and its effects on abundance of major species of ruminal bacteria

**DOI:** 10.1186/s40104-018-0230-8

**Published:** 2018-02-07

**Authors:** Mohamed K. Abdelmegeid, Ahmed A. Elolimy, Zheng Zhou, Vincenzo Lopreiato, Joshua C. McCann, Juan J. Loor

**Affiliations:** 1 Department of Animal Sciences, Mammalian NutriPhysioGenomics, University of Illinois, Urbana, IL 61801 USA; 2 Department of Animal Medicine, Faculty of Veterinary Medicine, Kafrelsheikh University, Kafr El-Shaikh, 33516 Egypt; 3 Department of Animal and Veterinary Sciences, Clemson University, Clemson, SC 29634 USA; 4 Department of Health Science, Interdepartmental Services Centre of Veterinary for Human and Animal Health, Magna Græcia University of Catanzaro, 88100 Catanzaro, Italy; 5 Division of Nutritional Sciences, Illinois Informatics Institute, University of Illinois, Urbana, IL USA

**Keywords:** Microbiome, Rumen microbes, Transition cow

## Abstract

**Background:**

Extensive degradation of amino acids in the rumen via microbial deamination decreases the post-ruminal availability of dietary indispensable amino acids. Together with the normal decrease in voluntary dry matter intake (DMI) around parturition in dairy cows, microbial metabolism contributes to a markedly negative balance of indispensable amino acids, including methionine which may be the first-limiting for milk production. The main objective of the current study was to profile changes in major bacterial species with key functions in cellulose and hemicellulose digestion, xylan breakdown, proteolytic action, propionic acid production, lactate utilization and ruminal biohydrogenation in cows supplemented with rumen-protected Methionine (SM; Smartamine M, Adisseo NA, Alpharetta, GA, USA) from −23 through 30 d relative to parturition. Because ~90% of the methionine in SM bypasses the rumen, ~10% of the methionine is released into the rumen and can be utilized by microbes.

**Results:**

As expected, there was an increase in overall DMI after parturition (Day, *P* < 0.05) during which cows consumed on average 19.6 kg/d versus 13.9 kg/d in the prepartum period. The postpartum diet contained greater concentrations of lipid and highly-fermentable carbohydrate from corn grain, which likely explains the increases in the relative abundance of *Anaerovibrio lipolytica*, *Megasphaera elsdenii*, *Prevotella bryantii*, *Selenomonas ruminantium*, *Streptococcus bovis*, and *Succinimonas amylolytica*. Despite similar DMI prepartum, cows fed SM had greater (Treatment × Day, *P* < 0.05) abundance prepartum of *Fibrobacter succinogenes, Succinimonas amylolytica*, and *Succinivibrio dextrinosolvens*. However, the greater DMI in cows fed SM after parturition (19.6 kg/d versus 13.9 kg/d) was associated with lower abundance of *Fibrobacter succinogenes* (2.13 × 10^−3^ versus 2.25 × 10^−4^) and *Selenomonas ruminantium* (2.98 × 10^−1^ versus 4.10 × 10^−1^). A lower abundance (Day, *P* < 0.05) was detected on d 20 compared with d −10 for *Fibrobacter succinogenes* and *Succinivibrio dextrinosolvens*. The relative abundance of *Butyrivibrio proteoclasticus* and *Eubacterium ruminantium* was stable across treatment and time.

**Conclusions:**

In diets with proper balance of rumen-degradable protein and fermentable carbohydrate, the small fraction of Methionine released from the rumen-protected supplement did not seem to compromise growth of major bacterial species in the rumen. In fact, it had a positive effect on 3 major species prepartum when DMI was similar between groups. Because the actual requirements of Methionine (and Lysine, for example) by the cow during the transition period are unknown, it appears warranted to study the rumen microbiome as it relates to supply of rumen-protected amino acids.

## Background

Extensive degradation of amino acids in the rumen [1] via microbial deamination [2, 3] substantially lowers the post-ruminal availability of dietary indispensable amino acids (IAA). Together with the normal decrease in voluntary dry matter intake (DMI) around parturition in dairy cows, microbial metabolism contributes to a markedly negative balance of IAA, including methionine which often is the first-limiting amino acid [4]. Therefore, the importance of enhancing the delivery methionine to the small intestine through “rumen by-pass” technologies has been underscored since the early 1960’s [5]. The physiologic impact of supplementing dairy diets with rumen-protected methionine (RPM) at lactation stages where needs are the greatest has received close attention worldwide, i.e. improving the overall health, metabolism, and production performance in dairy cows [6–9]. With the advances in methionine protection technology, a large proportion of protected methionine escapes ruminal degradation but a small fraction of methionine is still released into the rumen and may change the community composition of the rumen microbiota and their metabolism.

During the peripartal period, dairy cow diets move from higher-forage content before calving to higher-concentrate diets postpartum to provide the rumen microbial communities with more readily-available energy. As a result, microbial composition changes in favor of generating more volatile fatty acids (VFA) and microbial protein to serve as a fuel and amino acid sources, respectively, for body tissues and milk synthesis [10–12]. Therefore, nutritional management plays a key role in shaping the microbial ecosystem in the rumen [13, 14]. Despite the continued focus on nutritional management of peripartal cows, little is known about the response of rumen microbes to methionine supplementation. For example, Salsbury et al. [15] and Gil et al. [3] were among the first to observe that unprotected supplemental methionine enhanced ruminal bacteria growth rate and protein synthesis in vitro.

Although there is growing evidence that RPM enhances overall dairy cow health and productivity, whether ruminal bacteria composition changes in response to RPM supplementation remains unknown. Therefore, the objective of the current study was to profile changes in major bacterial species with key functions in cellulose and hemicellulose digestion, xylan breakdown, proteolytic action, propionic acid production, lactate utilization and ruminal biohydrogenation in cows supplemented with RPM (Smartamine M, Adisseo NA, Alpharetta, GA) from −23 through 30 d around parturition. Smartamine M is designed to release over 90% of the methionine along the small intestine. Thus, the remaining 10% of the methionine is released into the rumen and can be utilized by microbes. Hence, the hypothesis was that the liberated methionine portion into the rumen following dietary RPM supplementation could affect the composition of major ruminal bacteria around calving.

## Methods

Animal handling and all procedures of this study received approval from the Institutional Animal Care and Use Committee of the University of Illinois under protocol no. 13023. In total, a subset of 20 multiparous cows from a larger study [16] were randomly selected for ruminal fluid sampling. These cows were either fed a control diet without RPM (CON) or CON plus RPM supplementation (SM) at a rate of 0.08% of DMI (Smartamine M, Adisseo NA, Alpharetta, GA). Dosage of RPM was based on previous work from our research group [17]. At the start of the experimental feeding phase, none of the cows enrolled had received any type of RPM. All cows received a far-off diet (Table 1) as total mixed ration (TMR) from −50 to −22 d before expected calving date (1.40 Mcal/kg of DM, 10.2% rumen-degradable protein, and 4.1% rumen-undegradable protein), a close-up diet from −21 d to the expected calving date (1.52 Mcal/ kg of DM, 9.1% rumen-degradable protein, and 5.4% rumen-undegradable protein) and a lactation diet after calving through 30 d postpartum (1.71 Mcal/kg of DM, 9.7% rumen-degradable protein, and 7.5% rumen-undegradable protein). The TMR was delivered once daily (0600 h) using an electronic recognition feeding system for each cow (American Calan, Northwood, NH) before calving or in open individual mangers during lactation. The DM content of the TMR for the close-up and lactation diets was measured weekly for estimation of daily TMR dry matter offered. The required amount of RPM was calculated daily for each individual cow and was top-dressed from −21 ± 2 to 30 d relative to parturition once daily at the morning feeding using approximately 50 g of ground corn as carrier.

**Table 1:** Ingredients and chemical composition of experimental diets

	Diet		
Ingredient, % of DM	Far-off	Close-up	Lactation
Alfalfa silage	12.00	8.34	5.07
Alfalfa hay	–	4.29	2.98
Corn silage	33.00	36.40	33.41
Wheat straw	36.00	15.63	2.98
Cottonseed	–	–	3.58
Wet brewers grains	–	4.29	9.09
Ground shelled corn	4.00	12.86	23.87
Soy hulls	2.00	4.29	4.18
Soybean meal, 48% CP	7.92	2.57	2.39
Expeller soybean meal^a^	–	2.57	5.97
Soychlor^b^	0.15	3.86	–
Blood meal, 85% CP	1.00	–	–
ProVAAl AADvantage^c^	–	0.86	1.50
Urea	0.45	0.30	0.18
Rumen-inert fat^d^	–	–	1.02
Limestone	1.30	1.29	1.31
Salt	0.32	0.30	0.30
Dicalcium phosphate	0.12	0.18	0.30
Magnesium oxide	0.21	0.08	0.12
Magnesium sulfate	0.91	0.99	–
Sodium bicarbonate	–	–	0.79
Potassium carbonate	–	–	0.30
Calcium sulfate	–	–	0.12
Mineral vitamin mix^e^	0.20	0.17	0.18
Vitamin A^f^	0.015	–	–
Vitamin D^g^	0.025	–	–
Vitamin E^h^	0.38	0.39	–
Biotin	–	0.35	0.35

^a^SoyPLUS (West Central Soy, Ralston, IA)

^b^By West Central Soy

^c^Perdue AgSolutions LLC (Ansonia, OH)

^d^Energy Booster 100 (Milk Specialties Global, Eden Prairie, MN)

^e^Contained a minimum of 5% Mg, 10% S, 7.5% K, 2.0% Fe, 3.0% Zn, 3.0% Mn, 5,000 mg of Cu/kg, 250 mg of I/kg, 40 mg of Co/kg, 150 mg of Se/kg, 2,200 kIU of vitamin A/kg, 660 kIU of vitamin D_3_/kg, and 7,700 IU of vitamin E/kg

^f^Contained 30,000 kIU/kg

^g^Contained 5,009 kIU/kg

^h^Contained 44,000 kIU/kg

### Ruminal bacteria DNA isolation and qPCR amplification of 16S rDNA genes

At −10 d before expected calving date and d 20 postpartum, ruminal fluid was sampled from each cow using a stomach tube prior to the morning feeding. The sample was filtered through three layers of cheesecloth. Mixed ruminal fluid was immediately frozen in liquid nitrogen followed by storage at −80 °C. Total genomic DNA was isolated using the repeated bead beating method described by Yu and Morrison [18] for mechanical lysis of bacterial cell wall, employing the QIAamp DNA mini kit (QIAGEN) for DNA purification. The DNA quantity and quality were checked by 0.8% (wt/v) agarose gel electrophoresis and NanoDrop spectrophotometer (ND 1000, NanoDrop Technologies, Inc., Wilmington, DE, USA) at 260 nm. Extracted DNA was standardized to 8 ng/μL for qPCR.

The primer sets selected to amplify 10 targeted rumen bacterial species are listed in Table 2 and were validated using gel electrophoresis. A total of 10 μL of qPCR mixture contained 4 μL sample DNA, 5 μL 1× SYBR Green with ROX (Quanta BioSciences, Gaithersburg, MD, USA), 0.4 μL each of 10 μmol/L forward and reverse primers, and 0.2 μL DNase/RNase free water in a MicroAmp™ Optical 384-Well Reaction Plate (Applied Biosystems, Foster City, CA, USA). Negative controls without template DNA, standards, and samples were run on the same plate in triplicate. The qPCR reactions were performed with the ABI Prism 7900HT Fast Real-Time PCR System (Applied Biosystems, Foster City, CA, USA) using the following program: initial denaturation at 95 °C for 5 min, followed by 40 cycles of 1 s at 95 °C and 30 s annealing at 60 °C, except for eubacterial primer 3 that required an annealing temperature of 56 °C. A dissociation stage was performed to determine the specificity of the amplification. Relative abundance of bacterial species was calculated using the geometric mean of two universal primers [19, 20] with the efficiency-corrected Δ^−CT^ method [21]. Thus, the abundance of each target bacteria was determined relative to the overall abundance of the total bacteria as measured with the universal primers.

**Table 2:** Species-specific primers for the quantification of selected rumen bacterial populations using a real-time qPCR assay

Target bacterial species		Primer sequence (5' → 3')	Reference
*Anaerovibrio lipolytica*	F:^a^	GAAATGGATTCTAGTGGCAAACG	[12]
	R:^b^	ACATCGGTCATGCGACCAA	
*Butyrivibrio proteoclasticus*	F:	GGGCTTGCTTTGGAAACTGTT	[12]
	R:	CCCACCGATGTTCCTCCTAA	
*Eubacterium ruminantium*	F:	CTCCCGAGACTGAGGAAGCTTG	[37]
	R:	GTCCATCTCACACCACCGGA	
*Fibrobacter succinogenes*	F:	GCGGGTAGCAAACAGGATTAGA	[37]
	R:	CCCCCGGACACCCAGTAT	
*Megaspheara elsdenii*	F:	AGATGGGGACAACAGCTGGA	[37]
	R:	CGAAAGCTCCGAAGAGCCT	
*Prevotella bryantii*	F:	AGCGCAGGCCGTTTGG	[37]
	R:	GCTTCCTGTGCACTCAAGTCTGAC	
*Selenomonas ruminantium*	F:	CAATAAGCATTCCGCCTGGG	[37]
	R:	TTCACTCAATGTCAAGCCCTGG	
*Succinimonas amylolytica*	F:	CGTTGGGCGGTCATTTGAAAC	[29]
	R:	CCTGAGCGTCAGTTACTATCCAGA	
*Streptococcus bovis*	F:	TTCCTAGAGATAGGAAGTTTCTTCGG	[37]
	R:	ATGATGGCAACTAACAATAGGGGT	
*Succinivibrio dextrinosolvens*	F:	TAGGAGCTTGTGCGATAGTATGG	[29]
	R:	CTCACTATGTCAAGGTCAGGTAAGG	
Bacteria general 1	F:	GGATTAGATACCCTGGTAGT	[20]
	R:	CACGACACGAGCTGACG	
Bacteria general 2	F:	GTGSTGCAYGGYTGTCGTCA	[19]
	R:	ACGTCRTCCMCACCTTCCTC	

^a^*F* forward primer; ^b^*R* reverse primer

### Statistical analysis

Dry matter intake and relative abundance of bacteria were analyzed using the MIXED procedure of SAS 9.3 (SAS Inst. Inc., Cary, NC, USA). The fixed effects in the model were Day, Treatment, and the interaction of Day × Treatment. The random effect was cow within treatment.

## Results

### Dry matter intake

**Fig. 1:**
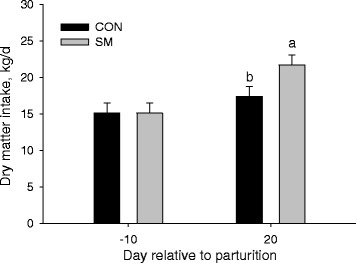
Dry matter intake in Holstein cows fed a control diet (CON) or CON supplemented with rumen-protected methionine (SM) from d −21 through d 30 relative to parturition. ^ab^Different letters denote treatment effects (*P* < 0.05) between treatments. The *P* value for the overall effect of Treatment, Day, and Treatment × Day was 0.002, 0.03, and 0.48, respectively. Bars indicate standard error of the means

As expected, there was an increase in overall DMI after parturition (Day, *P* < 0.05) during which cows consumed on average 19.6 kg/d versus 13.9 kg/d in the prepartum period. The overall effect of treatment (*P* < 0.05) was due to cows in the SM group consuming ~3 kg DM more than those in the CON group specifically after parturition (Fig. 1; 19.6 kg/d versus 13.9 kg/d).

### Abundance of ruminal bacteria

The relative abundance of target bacterial species is presented in Table 3. *Selenomonas ruminantium* was the only bacterial species with an overall treatment effect (*P* = 0.01) due to a 27.4% decrease in abundance with SM supplementation. Furthermore, this was the most-abundant bacterial species ranging from 2.63×10^−1^ at −10 d (SM) to 6.39×10^−1^ (a peak in abundance) at 20 d in cows fed CON.

**Table 3:** Relative abundance of microbial species in mixed ruminal fluid from Holstein cows fed a control diet (CON) or CON supplemented with rumen-protected methionine (SM) during the periparturient period

					Day × Treatment						
	Treatment		Day		CON		SM		*P* value^1^		
Species	CON	SM	−10	20	−10	20	−10	20	Trt	Day	T × D
*A. lipolytica*	3.28×10^−3^	3.42×10^−3^	2.50×10^-3b^	4.48×10^-3a^	2.25×10^−3^	4.77×10^−3^	2.77×10^−3^	4.21×10^−3^	0.82	<0.01	0.42
*B. proteoclasticus*	1.07×10^−2^	8.88×10^−3^	1.01×10^−2^	9.46×10^−3^	1.24×10^−2^	9.29×10^−3^	8.18×10^−3^	9.64×10^−3^	0.20	0.67	0.13
*E. ruminantium*	2.44×10^−2^	2.45×10^−2^	2.23×10^−2^	2.69×10^−2^	2.59×10^−2^	2.31×10^−2^	1.92×10^−2^	3.13×10^−2^	0.98	0.38	0.16
*F. succinogenes*	7.10×10^−4^	6.93×10^−4^	1.16×10^-3a^	4.25×10^-4b^	6.29×10^-4B^	8.02×10^-4B^	2.13×10^-3A^	2.25×10^-4C^	0.92	<0.01	<0.01
*M. elsdenii*	3.90×10^−5^	1.90×10^−5^	2.40×10^-6b^	2.34×10^-4a^	4.60×10^−6^	2.07×10^−4^	1.00×10^−6^	2.65×10^−4^	0.43	<0.01	0.24
*P. bryantii*	4.22×10^−3^	1.30×10^−3^	7.61×10^-4b^	7.20×10^-3a^	1.12×10^−3^	1.60×10^−2^	5.19×10^−4^	3.25×10^−3^	0.12	<0.01	0.57
*S. ruminantium*	4.10×10^-1a^	2.98×10^-1b^	2.63×10^-1b^	4.65×10^-1a^	2.63×10^-1C^	6.39×10^-1A^	2.63×10^-1C^	3.37×10^-1BC^	0.01	<0.01	0.01
*S. amylolytica*	1.02×10^−4^	2.30×10^−4^	2.51×10^−4^	9.40×10^−5^	9.90×10^-5B^	1.05×10^-4B^	6.32×10^-4A^	8.40×10^-5B^	0.16	0.07	0.05
*S. dextrinosolvens*	9.10×10^−5^	1.60×10^−4^	1.71×10^-4a^	8.50×10^-5b^	8.10×10^-5B^	1.02×10^-4B^	3.64×10^-4A^	7.00×10^-5B^	0.10	0.05	0.01
*S. bovis*	8.09×10^−3^	3.50×10^−3^	3.17×10^-3b^	8.93×10^-3a^	6.68×10^−3^	9.79×10^−3^	1.51×10^−3^	8.14×10^−3^	0.22	0.02	0.14

^1^*Trt* treatment effect, *Day* day effect, *T × D* Treatment by Day interaction

^ab^Means for overall Treatment or Day effect differ (*P* < 0.05)

^A-C^Means for the interaction of Day × Treatment differ (*P* < 0.05)

Concerning day effects, greater relative abundance of *Fibrobacter succinogenes* (*P* < 0.01) and *Succinivibrio dextrinosolvens* (*P* = 0.05) was observed at −10 d compared with 20 d postpartum. Furthermore, there was a day effect (*P* < 0.01) for *Anaerovibrio lipolytica, Megasphaera elsdenii, Prevotella bryantii*, *Selenomonas ruminantium* and *Streptococcus bovis* (*P* = 0.02) as these bacterial species had greater abundance at 20 d compared with −10 d around parturition. *Succinimonas amylolytica* tended to be greater (*P* = 0.07) at −10 compared with 20 d.

The relative abundance of *Butyrivibrio proteoclasticus* and *Eubacterium ruminantium* was stable (*P* > 0.05) across treatment and time. However, *Eubacterium ruminantium* was the second most-abundant bacterial species among those studied, ranging from 1.92×10^−2^ at −10 d with SM to 3.13×10^−2^ at 20 d also with SM.

A Day × Treatment interaction was observed for *Fibrobacter succinogenes, Selenomonas ruminantium*, *Succinivibrio dextrinosolvens,* and *S. amylolytica. Selenomonas ruminantium* abundance nearly doubled (*P* = 0.01) between −10 and 20 d only in CON cows*.* In contrast, *Fibrobacter succinogenes, Succinimonas amylolytica* and *Succinivibrio dextrinosolvens* decreased on d 20 in SM cows while no change or a decrease was observed in CON cows*.*

## Discussion

*Selenomonas ruminantium* was the most abundant bacterial species observed in the present study and agrees with previous classical studies indicating that this Gram-negative bacteria could account for up to 51% of the total viable bacterial counts within the rumen [22]. The fact that *Selenomonas ruminantium* was overall 27.4% lower in response to RPM supplementation could have been associated with the greater DMI in those cows after parturition. This microbial species is a propionate-producer through decarboxylation of succinate, which involves lactic acid production particularly during feeding of higher-concentrate diets [23, 24]. Thus, because the numbers of *Selenomonas ruminantium* in SM-fed cows actually increased numerically after parturition relative to the prepartum period, it is likely that the greater DMI in those cows either diluted the numbers of this species or increased the diversity of the rumen population.

Availability of propionate for gluconeogenesis by the animal during the transition into lactation relies heavily on both *Selenomonas ruminantium* and *Megasphaera elsdenii* numbers in the rumen [25]. Approximately 90% of glucose in ruminants is supplied by gluconeogenesis, with 50 to 60% of this being derived from propionate [26]. Thus, the longitudinal change in *Selenomonas ruminantium* and *Megasphaera elsdenii* agrees with higher content of rapidly-fermentable carbohydrate in the postpartum diet, i.e. substrate availability likely helped enhance the numbers of these species [23].

Except for *Butyrivibrio proteoclasticus* (fibrolytic species), the greater numbers of *Anaerovibrio lipolytica, Prevotella bryantii, Megasphaera elsdenii, Selenomonas ruminantium,* and *Streptococcus bovis* at d 20 postpartum along with the lower numbers of *Fibrobacter succinogenes, Succinimonas amylolytica*, and *Succinivibrio dextrinosolvens* were consistent with a previous study [12]. They attributed these changes to the important features associated with the transition into lactation, e.g. greater concentration of lipid and rapidly-fermentable carbohydrate as a way to provide more energy for the cow during a period when voluntary DMI may be less than optimal.

The fact that abundance of *Streptococcus bovis* and *Prevotella bryantii* at 20 d postpartum was associated with greater abundance of *Selenomonas ruminantium* and *Megasphaera elsdenii* seems to indicate some degree of synchronization. These results are broadly consistent with other studies demonstrating that, as dietary grain increases, the prevalence of starch-fermenting bacteria like *Streptococcus bovis* is increased with a consequent synchronized increase in the population ratio of lactate-consuming bacteria like *Selenomonas ruminantium* and *Megasphaera elsdenii* to help eliminate lactate via fermentation to propionate [25, 27–29]. *Prevotella* spp. grows rapidly on starch and produce succinate and propionate as major end-products [29, 30]. A previous study [12] attributed a higher proportion of *Megasphaera elsdenii* and *Prevotella bryantii* after parturition to the higher DMI, which agrees with data in the present study.

The lower abundance of *Fibrobacter succinogenes* postpartum was associated with greater abundance of *Anaerovibrio lipolytica* and seems to be partly explained by a potential negative effect of higher availability of dietary unsaturated fatty acids on ruminal cellulolytic bacteria [31]. Indirectly, these results suggest that the greater fiber content of the prepartum diet allowed for greater numbers of *Fibrobacter succinogenes*, which agrees with a previous study [25]. In ruminants fed high-forage diets *Fibrobacter succinogenes* is one of the predominant cellulolytic species, favoring the greater production of acetate relative to propionate [32].

The greater abundance of fibrolytic bacteria such as *Fibrobacter succinogenes,* and starch-degrading bacteria such as *Succinimonas amylolytica* and *Succinivibrio dextrinosolvens* at −10 d relative to parturition in the SM group was not associated with differences in DMI; however, the lower abundance of these species in response to SM at 20 d could have been related with the greater DMI in those cows, potentially due to a change in the rumen kinetics such as liquid dilution rate. It is well-established that increases in dietary concentrate/grain to forage not only increase intake but also liquid dilution rate [33]. Thus, besides the greater DMI in cows fed SM, the higher content of corn grain in the postpartum diet (Table 1) might have reduced rumen-retention time of digesta which could partly explain the lower abundance of bacteria. Recent studies demonstrated that RPM can increase DMI both pre and postpartum in dairy cows [16, 34]. It remains to be determined if the greater DMI and lower relative abundance due to feeding SM would affect digestive enzyme activity within the rumen.

The response of *Anaerovibrio lipolytica* after parturition confirms its role in the hydrolysis of triacylglycerol into free fatty acids in the rumen [35]. The inverse relationship between *Anaerovibrio lipolytica* and *Fibrobacter succinogenes* as it relates to dietary fiber and lipid level was confirmed previously [36]. A previous study [12] also speculated that *Anaerovibrio lipolytica* (like *Megasphaera elsdenii*) can use lactate as a substrate for growth during feeding of higher-fermentable diets after parturition. The lack of day, treatment, or interaction effect on the relative abundance of *Butyrivibrio proteoclasticus* and *Eubacterium ruminantium* implies that these bacteria might be less sensitive to changes during the transition period.

Clearly, the diets used in the present study appear to have provided enough fermentable energy and nitrogen sources to allow normal growth of the major microbes studied. Whether the rumen microbiome responds to the supply of rumen-protected supplements needs to be explored further. This is particularly important because the actual requirements of methionine (and lysine, for example) during the transition period are unknown.

## Conclusions

In diets with proper balance of rumen-degradable protein and fermentable carbohydrate, the small fraction of Methionine released from the rumen-protected supplement did not seem to compromise growth of major bacterial species in the rumen. In fact, it had a positive effect on 3 major species prepartum when DMI was similar between groups. Because the actual requirements of Methionine (and Lysine, for example) by the cow during the transition period are unknown, it appears warranted to study the rumen microbiome as it relates to supply of rumen-protected amino acids.

## Abbreviations

DM: Dry matter
DMI: Dry matter intake
IAA: Indispensable amino acids
RPM: Rumen-protected methionine
TMR: Total mixed ration
VFA: Volatile fatty acid
